# Circulating miR-122 and miR-200a as biomarkers for fatal liver disease in ART-treated, HIV-1-infected individuals

**DOI:** 10.1038/s41598-017-11405-8

**Published:** 2017-09-07

**Authors:** Daniel D. Murray, Kazuo Suzuki, Matthew Law, Jonel Trebicka, Jacquie Neuhaus Nordwall, Margaret Johnson, Michael J. Vjecha, Anthony D. Kelleher, Sean Emery

**Affiliations:** 1 The Kirby Institute for Infection and Immunity in Society, University of New South Wales, Sydney, Australia; 2 Department of Internal Medicine, University of Bonn, Bonn, Germany; 3 University of Minnesota, Minneapolis, Minnesota United States of America; 4 Ian Charleson Day Centre, Royal Free Hampstead NHS Trust, London, United Kingdom; 5 Institute for Clinical Research, Veterans Affairs Medical Center, Washington D.C., United States of America; 6 Faculty of Medicine, University of Queensland, Brisbane, Australia

## Abstract

Liver disease is one of the main contributors to the increased levels of morbidity and mortality seen in the HIV-1-infected, ART-treated population. Circulating miRNAs, particularly those located inside extracellular vesicles, are seen as promising biomarkers for a number of human disease conditions, including liver-related diseases. Here, we show that serum levels of miR-122 and miR-200a are greater in HIV/HCV co-infected individuals compared to HIV-1 mono-infected individuals. We also show that miR-122 and miR-200a are elevated in ART-treated, HIV-1-infected individuals prior to the development of fatal liver disease, suggesting that these miRNA may have some potential clinical utility as biomarkers. While this study is hypothesis generating, it shows clearly that both miR-122 and miR-200a are promising novel biomarkers for liver disease in the ART-treated, HIV-1-infected population.

## Introduction

HIV-1 infection remains a critical, unsolved, global health problem despite the introduction of effective anti-retroviral therapies (ART), which have decreased both morbidity and mortality, particularly in the developed world. However, there is currently no cure, no vaccine, and treatment remains life-long. Furthermore, individuals on long term ART have increased rates of morbidity and mortality compared to uninfected individuals, and these differences appear related to a collection of end organ diseases that collectively termed Serious Non-AIDS Events (SNAEs)^1–4^. Liver disease is one of the main causes of SNAEs and is associated with the highest rates of mortality of all SNAEs^5, 6^. A major factor contributing to liver disease in HIV-1-infected individuals is HCV co-infection. Despite treatment with ART, co-infection with HCV drastically increases the risk of the development of, and mortality due to SNAEs^5–7^. While new direct-acting antiviral drugs (DAAs) against HCV have reduced the incidence of HIV/HCV co-infection, high and sustained rates of treatment uptake have not yet been achieved in most jurisdictions. Additionally, DAAs give no protection from re-infection and have no effect on other causes of chronic liver disease. Therefore, despite the development of DAAs, liver disease, both HCV and non-HCV-related, will remain a critical health priority. Therefore, identification of predictive biomarkers of liver disease would allow the development of more informative risk assessment tools for HIV-1 positive patients.

MicroRNAs (miRNAs) are small non-coding RNAs that are important post-transcriptional regulators of gene expression. They act upon complementary strands of mRNA inhibiting protein production in the cytoplasm. In addition to their cellular function, miRNAs have also recently been found to be relatively abundant in the circulation. These circulating miRNAs are increasingly being found to play important roles in intercellular communication and hold promise as non-invasive biomarkers in a number of disease conditions^8^. MiRNAs in the circulation are extremely stable and are thought to be able to avoid RNase activity through two mechanisms: first, by associating with Argonaute (AGO) proteins^9, 10^; and second, by being secreted in extracellular vesicles (EVs)^10, 11^. Although it seems likely that the secretion of miRNAs into EVs is also dependent on AGO proteins^12^.

In our previous study of 126 cases, who died whilst on ART and 247 matched controls, we measured a panel of 21 miRNAs, chosen based on published associations with human disease conditions that constitute SNAEs^13^. These miRNAs, when measured in serum, showed no associations with mortality (all-cause, cardiovascular or malignancy related). In this post-hoc study we further analysed these groups, to determine if these miRNAs associated with liver-related morbidity and mortality.

This new analysis showed that circulating levels of miR-122 and miR-200a were elevated in HIV/HCV co-infected individuals, compared to HIV mono-infected individuals. Additionally, we observed greater pre-ART levels of circulating miR-122 and miR-200a, compared to matched controls, in HIV-1 positive individuals who died from liver-related diseases whilst undergoing suppressive ART. Overall, the data presented here show that both miR-122 and miR-200a are promising biomarkers, whose increase may precede the development of severe liver disease in the ART-treated HIV-1-infected population.

## Results

### Study Population

Participants were selected from the control arms of the SMART^14^ and ESPRIT^15^ trials, that is those receiving continuous ART. All participants in these trials were HIV-1 positive. Samples analysed in this study were taken at baseline, prior to the initiation of ART. Cases were defined as those participants who died from any cause during follow up. Controls were defined as participants who were known to be alive at completion of follow up and matched for age, gender, location (continent) and randomisation date (±3 months). For full details on all the cases and controls, including baseline characteristics, see Supplemental Table 1.

#### Liver case and control selection

The 13 liver cases, and their 25 matched controls, were selected from the individuals analysed in the previous study, which investigated the associations between a panel of circulating miRNAs and all-cause mortality^13^. Liver cases were defined as those that died from liver-related causes, including Hepatitis C, Hepatitis B or non-viral liver failure (See Table 1 for full details), whilst undergoing suppressive ART. All the liver cases that died from HCV related causes had one matched control that was HCV positive and one that was HCV negative. Full baseline characteristics for the liver cases and controls can be found in Table 2.

**Table 1:** Causes of Death for the Liver Cases.

Cause of Death	Number	%
HCV with cirrhosis	4	30
HCV with liver failure	1	8
HCV with liver cancer	1	8
HBV with cirrhosis	2	15
HBV with liver failure	1	8
HBV with liver cancer	1	8
Liver failure (other than HCV and HBV Liver failure)	3	23

**Table 2:** Baseline Characteristics for the Liver Cases and their matched Controls.

Baseline Characteristics	Case (n = 13)	Control (n = 25)
Age ± SD (years)	48.69 ± 9.48	46.88 ± 8.07
Mean baseline CD4 + T cell count (cells/µl) ± SD	487.9 ± 156.8	582.6 ± 243.2
Mean nadir CD4 + T cell count (cells/µl) ± SD^a^	155.6 ± 109.0	278.2 ± 278.2
Mean BMI (kg/m^2^) ± SD	22.08 ± 4.32	23.52 ± 3.31
Mean hs-CRP (mg/L) ± SD^a^	9.95 ± 17.98	2.58 ± 4.01
D-dimer (µg/mL)	0.43 ± 0.32	0.37 ± 0.27
IL-6 (pg/mL)^a^	3.71 ± 1.92	2.27 ± 1.39
Time to death^b^ (days)	1022 ± 660.9	2080 ± 585.3
Gender - no. (%) male	11 (85)	21 (84)
Race - no. (%) white	9 (70)	16 (64)
Race - no. (%) black	1 (7)	2 (8)
Race - no. (%) other/unknown	3 (23)	7 (28)
HBV - no. (%) surface antigen positive	2 (15)	0 (0)
HCV - no. (%) antibody positive	4 (31)	5 (20)
Previous AIDS - no. (%)	3 (23)	8 (28)
Diabetes - no. (%) baseline positive	1 (7)	2 (8)
CVD - no. (%) baseline positive	0 (0)	0 (0)
Lipid - Lowering Drug no. (%) baseline positive)	2 (15)	3 (12)
Blood Pressure Lowering Drug - no. (%) baseline positive	2 (15)	1 (4)

^a^Significantly different between cases and controls, as measured by Mann-Whitney U test.

^b^Time to death is days from randomization to death for the cases and days from randomization to censoring for the controls.

### Serum levels of miR-122 and miR-200a were greater in ART-treated HIV/HCV co-infected individuals and correlate with AST and ALT levels

**Figure 1:**
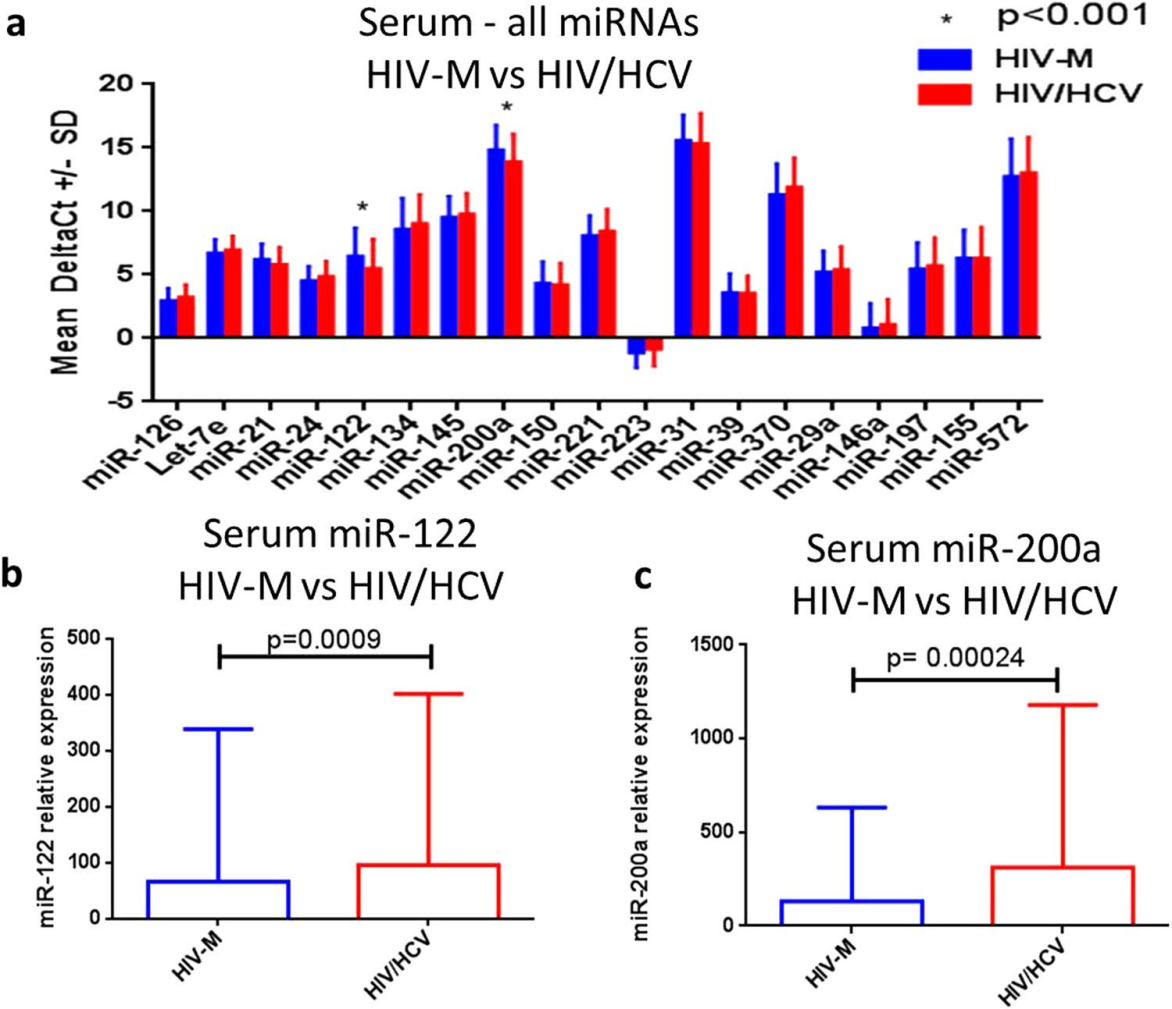
Expression of serum miRNAs in HIV/HCV co-infected individuals. The mean DeltaCT (miR of interest – miR-16) of all 19 miRNAs were compared between the HIV/HCV group and the HIV-M group using a multiple-t test with Sidak-Bonferroni correction (**a**). The relative expression of the two significantly different miRNAs between the two groups is also plotted (**b** and **c**).

Levels of the 21 circulating miRNAs were analysed, from the 373 cases and controls, to determine if there were any differences between HIV/HCV co-infected individuals (HIV/HCV) (n = 82) vs HIV-mono-infected individuals (HIV-M) (n = 291), regardless of case or control status. The levels of miR-122 (p = 0.0009) and miR-200a (p = 0.00024) were significantly greater in the HIV/HCV group compared to the HIV-M group (Fig. 1). Both these values were still significant when adjusted for multiple testing, using the Sidak-Bonferroni method (corrected p value 0.0026). The levels of the 19 other miRNAs, analysed in our previous study looking into the associations between a panel of miRNAs and mortality (all-cause, CVD and cancer)^13^, were not significantly different between the HIV-M and HIV/HCV groups (Fig. 1a).

To determine whether these miRNAs showed any association with liver injury, we compared the levels of miR-122 and miR-200a with the available baseline levels of aminotransferases AST (n = 179) and ALT (n = 181) (Supplemental Fig. 1). Both miR-122 and miR-200a showed modest, but statistically significant positive correlations with AST (r = 0.37 & p < 0.0001 and r = 0.29 & p = 0.0003 respectively) and ALT (r = 0.42 & p < 0.0001 and r = 0.33 and p < 0.0001 respectively).

### MiR-122 and miR-200a were greater in the serum of individuals who develop fatal liver disease

**Figure 2:**
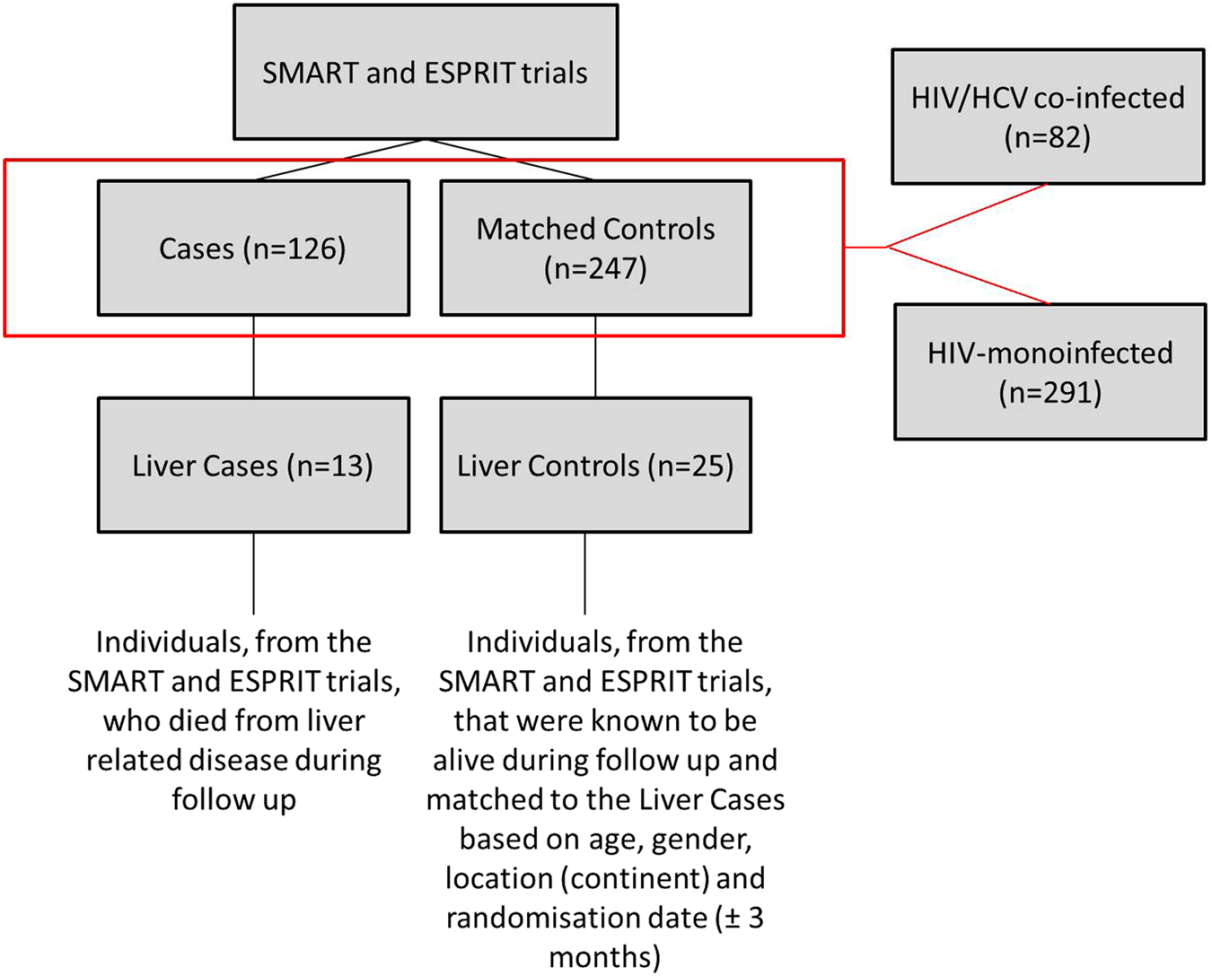
Selection of Liver cases and controls from the SMART and ESPRIT studies. 13 of the original 126 cases died from liver-related diseases. These 13 cases had 25 matched controls. Of the original 373 cases and matched controls, 82 were HIV/HCV co-infected while 291 were HIV-monoinfected.

We next analysed whether the expression of miR-122 and miR-200a were differentially regulated in individuals who died from liver-related diseases. Of the 126 cases analysed in our previous study^13^, 13 died from liver-related causes. The process of selection for these Liver Cases and Controls can be found in Fig. 2. On average, the death due to liver disease occurred 1022 ( ± 660) days after baseline.

**Figure 3:**
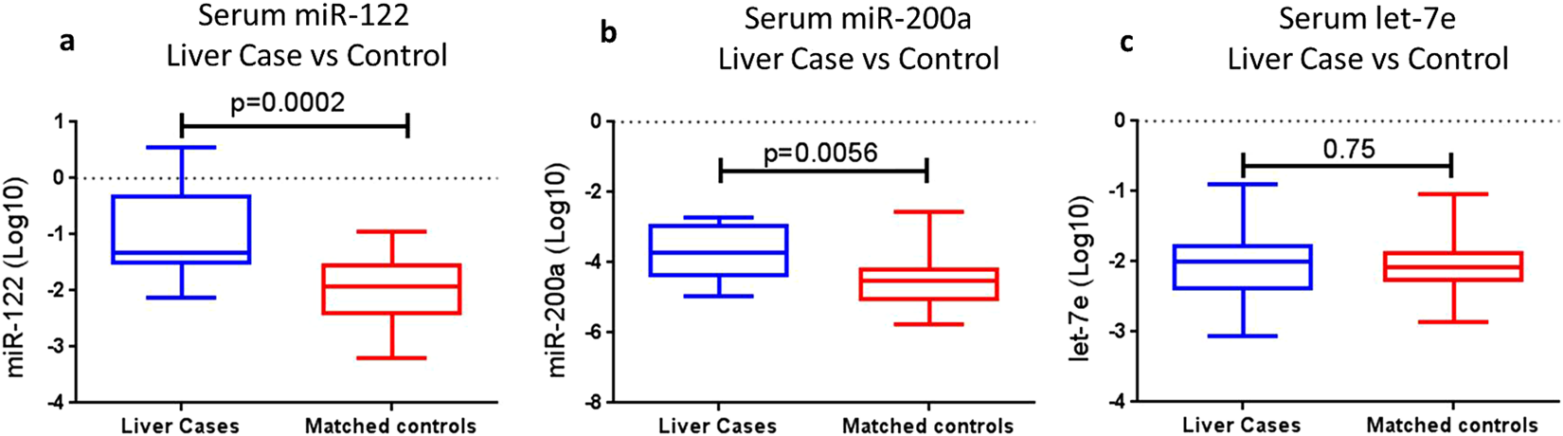
Serum levels of miR-122, miR-200a and let-7e in liver cases and controls. Relative expression (normalised to miR-16) of (**a**) miR-122, (**b**) miR-200a and **c**) Let-7e were log10 normalised and plotted for the Liver Cases (n = 13) and their matched controls (n = 25). Differences were measured using a Mann-Whitney U test with differences deemed significant with p < 0.05.

At study entry (i.e. before ART initiation), serum levels of both miR-122 (p = 0.0002) and miR-200a (p = 0.0056), but not let-7e (a miRNA that showed no difference between the HCV/HIV and HIV-M groups), were significantly greater in the liver cases compared to their matched controls. A 2 and 1.22 fold difference between means for miR-122 and miR-200a, respectively, were observed (Fig. 3a and b). There was no difference in let-7e levels (p = 0.75) between the two groups (Fig. 3c).

### MiR-122 and miR-200a were greater in PEG-precipitated particles of individuals who develop fatal liver disease

**Figure 4:**
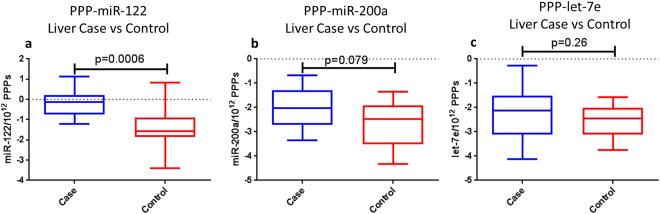
Levels of PPP-associated miR-122, miR-200a and let-7e in liver cases and controls. The levels of (**a**) miR-122, (**b**) miR-200a and (**c**) let-7e were measured in the PPPs of the liver cases and controls. miRNA levels were normalised to cel-miR-39 and divided by PPP concentration to give an estimation of relative amount of miRNA per 10^12^ PPPs. miR-122 was clearly increased in liver cases (n = 13) compared to controls (n = 25) and miR-200a also approached significance showing a clear trend for increase in liver cases (n = 11) compared to controls (n = 20). Let-7e was no different between liver cases (n = 11) and controls (n = 20). Not all PCRs were successful at amplifying the miRNAs so the full complement of cases and controls were not always available for the analyses. Differences were measured using a Mann-Whitney U test with differences deemed significant with p < 0.05.

In order to determine whether miR-122 and miR-200a were present in the circulation packaged in EVs, we precipitated the serum using the PEG-based ExoQuick reagent. While ExoQuick reagent is effective at purifying EVs, we could rule out contamination with other circulating particles (i.e. lipoproteins and protein complexes), therefore particles purified in this manner will be referred to as PEG-precipitated particles (PPPs). Once the PPPs were purified, they were then quantified using Nanoparticle Tracking Analysis, on the Malvern NanoSight. RNA was then extracted, before RTqPCR was carried out for miRs-122, miR-200a, let-7e and the spike-in control cel-miR-39, using individual qPCR probes and pre-amplified cDNA. PCRs for miR-200a and let-7e either failed or were considered unreliable in 7/38 individuals while miR-122 failed in 5/38 individuals. These samples were not included in further analysis. In the purified PPPs, miR-122 was significantly greater in the liver cases compared to their matched controls (p < 0.01) with a 3.3 fold difference observed the mean of liver cases compared to controls (Fig. 4a). PPP levels of miR-200a, while not statistically significant (p = 0.079), were much greater in the mean of liver cases compared to controls (4.5 fold difference) (Fig. 4b). No differences were observed in the levels of let-7e (p = 0.297) (Fig. 4c). Importantly, no differences were observed between liver cases and controls for PPP concentration (Supplemental Fig. 2) or PPP size (Supplemental Fig. 3). Additionally, PPP concentration did not correlate with miRNA levels that were normalised only to cel-miR-39 (Supplemental Fig. 4).

### PPP-associated miR-122 and miR-200a correlate with levels of AST and ALT

**Figure 5:**
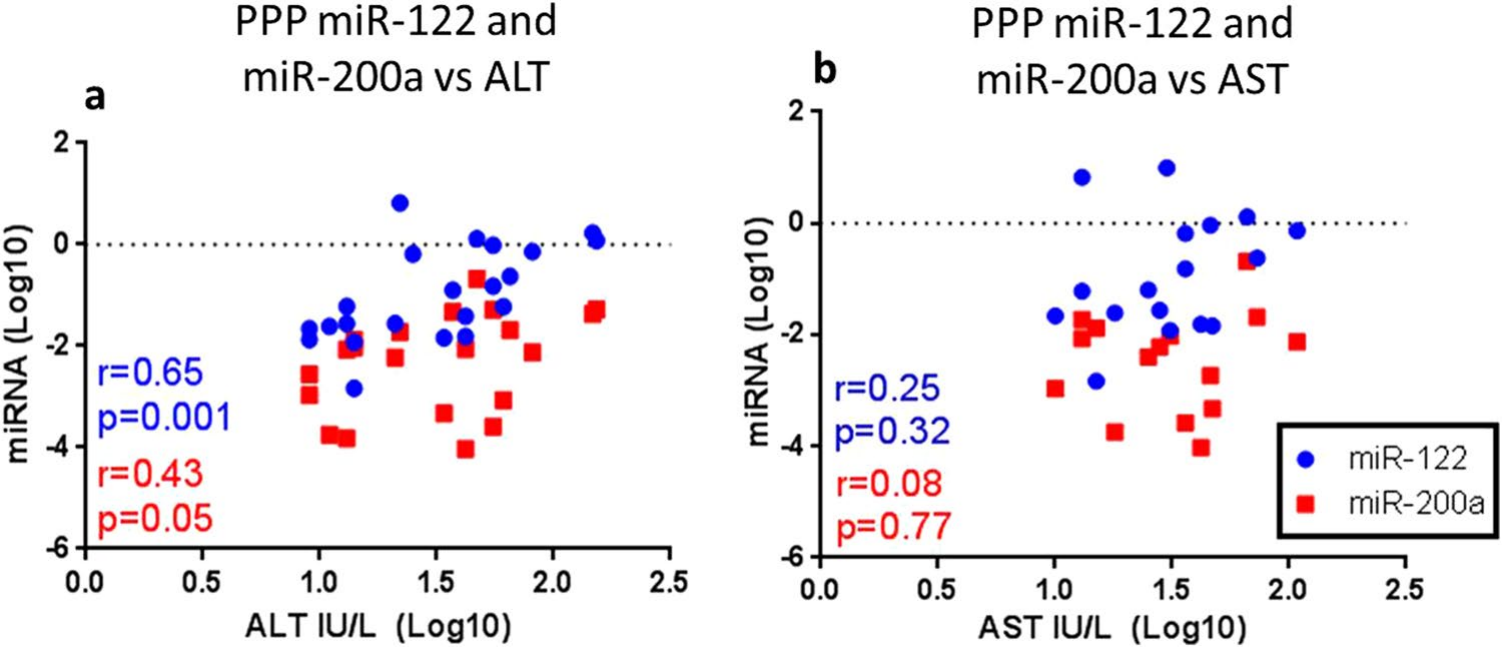
Correlations of PPP-associated miRNAs with ALT and AST. Relative expression (normalised to cel-miR-39 and PPP number) of miR-122 and miR-200a, measured in the PPPs of the Liver cases and controls and controls from the SMART and ESPRIT studies, (**a)** ALT and (**b)** AST were log normalised and plotted. Correlations were analysed using Spearman’s non-parametric correlation coefficient with a relationship deemed significant with p < 0.05. XY pairs were available for 22 individuals in the miR-122 vs ALT analysis, 21 for miR-200a vs ALT, 17 in the miR-122 vs AST analysis, 15 for miR-200a vs AST.

Next the levels of PPP-associated miRNAs were correlated with AST and ALT of the liver cases and controls. Only miR-122 significantly correlated with ALT (r = 0.65, p = 0.001) (Fig. 5a) while miR-200a was on the cusp of significance (r = 0.43, p = 0.05) (Fig. 5b). No correlation was observed for let-7e (Fig. 5c). Additionally, none of the miRNA correlated with AST levels (Fig. 5). Matching PCR results and ALT levels were available for 22/38 individuals for miR-122, 21/38 for miR-200a and 19/38 for Let-7e, while matching AST results were available for 17/38 individuals for miR-122, 15/38 for miR-200a and 17/38 for let-7e.

### Correlation of miR-122 and miR-200a with IL-6

**Figure 6:**
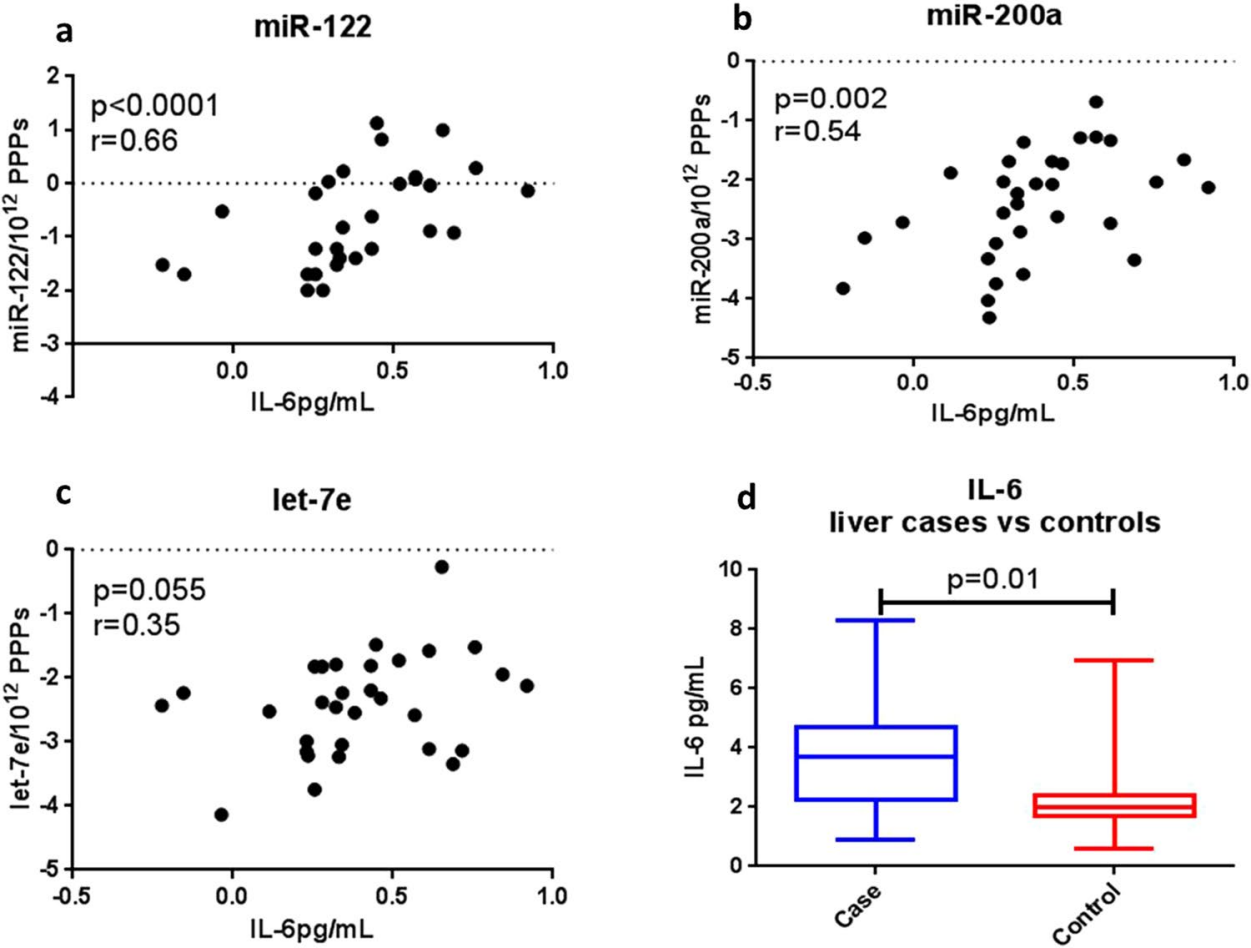
Correlations of PPP-associated miRNAs with IL-6. Relative expression of the miRNAs measured in the PPPs, normalised to CEL-miR-39 and PPP count, and IL-6 were log transformed and correlated using the nonparametric Spearman’s Correlation co-efficient. Data was considered significant with a p value < 0.05. (**a**) miR-122 and (**b**) miR-200a both significantly correlated with IL-6 while (**c**) the association with let-7e was not significant, but approached significance. (**d**) Levels of baseline IL-6 were significantly increased in liver cases compared to controls. Matching xy pairs were available for 30 individuals in the miR-122 analysis and 31 individuals in the miR-200a and let-7e analyses.

Both miR-122 and miR-200a correlated with IL-6 in the samples analysed in our previous study investigating the associations of these miRNAs with all-cause mortality^13^. This relationship was maintained when we compared levels of IL-6 with the three miRNAs measured in the PPPs. As expected PPP-associated miR-122 (p = 0.0001, r = 0.66) (Fig. 6a) and miR-200a (p = 0.002, r = 0.54) (Fig. 6b) correlated with the IL-6 measurements but not let -7e (Fig. 6c). Levels of IL-6 were significantly greater in the liver cases vs controls (Fig. 6d). No significant correlations were observed when levels of PPP- associated miR-122 and miR-200a were compared to d-dimer (p = 0.5 and 0.9 respectively) and hs-CRP (p = 0.1 and 0.24 respectively).

## Discussion

The potential of circulating miRNAs to act as biomarkers for liver disease in the ART-treated HIV-1-infected population has yet to be thoroughly explored. However, the data presented above indicates a great deal of potential for miR-122 and miR-200a. The expression of both of these miRNAs was greater in the serum of HCV/HIV co-infected individuals, compared to HIV-1 mono-infected individuals. Additionally, serum levels of both these miRNA correlated modestly with the liver aminotransferases ALT and AST, current biomarkers for liver injury, as well as IL-6^13^, a marker of systemic inflammation and adverse outcome on ART^16, 17^. The levels of these two miRNAs were then analysed in individuals who developed fatal liver diseases whilst being treated for HIV-1 infection. In the serum of these liver cases miR-122 (p = 0.0002, fold change = 2) and miR-200a (p = 0.0056, fold change = 1.22) were modestly, but significantly elevated in liver cases compared to controls, while let-7e, a miRNA that was not observed to be elevated in HCV/HIV co-infection and did not correlate with AST/ALT levels, was unchanged. Furthermore, in the PPPs of these liver cases and controls, the expression of miR-122 was significantly greater (p < 0.01, fold change = 3.3) and miR-200a showed a clear trend towards significance (p = 0.079, fold change = 4.5) in the liver cases compared to their matched controls, while, as expected, let-7e, which showed no association with liver disease in serum, showed no difference in the PPPs.

One interesting observation was that the differences between levels of miR-122 and miR-200a in liver cases and controls were much greater when serum was precipitated using ExoQuick reagent. While the contribution of EV-free, AGO-associated miRNAs cannot be ruled out, this observation, along with the observation that there was no difference in size or number of particles between liver cases and controls, suggests that the observed increase in miR-122 and miR-200a is due to a release of these miRNAs in PPPs. It is well known that ExoQuick and other PEG-based precipitation methods are not as efficient at extracting pure populations of EVs, compared to ultracentrifugation based methodologies. However, PEG based methodologies are relatively simple and do not require any expensive, machines – such as ultracentrifuges. As the burden of HIV-related disease remains in resource limited settings, much simpler technologies - such as PEG based reagents – may represent a tractable methodology to increase the EV purity enough to increase the clinical utility of circulating miRNA biomarkers. It is clear that future studies will have to fully characterise the location of circulating miR-122 and miR-200a in the context of HIV related liver disease. Yet, future studies may benefit from further exploring the clinical utility of miR-122 and miR-200a when extracted from both serum and PEG precipitated serum – with and without the use of technologies such as the NanoSight– as these methodologies may be more easily adopted in resource limited settings.

Previous studies have reported that expression of both miR-122 and miR-200a are dysregulated in the circulation during liver disease^18^. The greater expression of miR-122, the most highly expressed miRNA in hepatocytes^19^, is not surprising as this miRNA has been found to be elevated in HIV/HCV co-infected individuals previously^20, 21^. Additionally, miR-122 has been found, associated with HCV RNA, in the exosomes of HCV mono-infected individuals^22, 23^. Importantly out study is the first to associate elevated levels of miR-122 and miR-200a, at baseline, with the development of fatal liver disease in the future. On average the death due to liver disease occurred 1022 days after the miRNAs were elevated, however, the standard deviation was quite large (660 days) in the cases. This shows that these miRNAs may not just associate with current liver disease, but may be useful in predicting future adverse outcomes due to liver disease. However, the sample size analysed in this study was small and would need to be increased significantly, and reproduced in another cohort before we could reliably estimate hazard ratios for predicting mortality due to liver disease.

It is important to note that the vast majority of miRNAs are expressed in a multitude of cellular sources. Usually, circulating miRNA analysis does not distinguish between miRNAs of different cellular sources. Further, because it reflects an aggregation of sources, measuring bulk levels may miss perturbations of production from one source if these are balanced by changes in the production or uptake of the same miRNA by another source. However, miR-122 is almost exclusively expressed in hepatocytes and its dysregulation in the circulation is almost certainly due to its release from these cells^19^. MiR-200a, on the other hand, is not exclusively found in the liver - other sources include kidney and lung tissue^24–26^. Yet, expression of miR-200a is significantly enriched in hepatocytes and its similar expression profile to miR-122 in this study suggests that changes in its expression in the circulation are due to its release from hepatocytes^24–28^.

The data presented above clearly shows that miR-122 and miR-200a are greater in the circulation during liver disease. However, it is not clear how and why these miRNAs are being segregated into these vesicles. In order to elucidate the purpose of the release of these miRNAs it is essential to consider the role of these miRNAs in tissues. In a cellular context miR-200a is decreased in hepatocellular carcinoma (HCC), a disease in which hepatic cells avoid apoptosis, but increased in diseases characterised by hepatocyte cell death, such as fibrosis and Non-alcoholic fatty liver disease, suggesting a role for these miRNA in hepatic cell survival^27–30^. Additionally, miR-200a has been shown to be induced by the tumour suppressor p53 to mediate apoptosis in liver disease^31^, and can target a number of anti-apoptotic and oncogenic proteins such as EGRF and hepatocyte growth factor receptor (c-Met) in non-small cell lung cancer^32^, as well as Vasohibin 2, a protein that promotes angiogenesis and invasion, in HCC^33^. The cellular function of miR-122 is more difficult to ascertain, as this miRNA is associated with a wide array of potential cellular targets during liver disease (reviewed in ref. 34). However, miR-122 is also down-regulated in HCC and has been associated with disease progression and anti-apoptotic pathways in HCC cells^35–39^. Overall, the cellular data suggest that miR-122 and miR-200a may play a role in hepatocyte survival.

The correlations of miR-122 and miR-200a with IL-6 are interesting, as IL-6 is a marker of systemic inflammation, and it’s increase is associated with an increased risk of the development of SNAEs^16, 17^. In the literature, IL-6 signalling has been shown to promote hepatocyte proliferation and survival^40–45^ and IL-6 expression is increased in a number of liver specific diseases including HBV and HCV infection^40, 46^. Therefore, it may be that one of the mechanisms in which IL-6 promotes hepatocyte proliferation and survival is by jettisoning, in EVs, these two potentially apoptotic miRNAs. However, what is not clear from these cellular studies is if the elevated levels of miR-122 and miR-200a, seen in our study, are contributing to the increase in IL-6, or are merely a down-stream result of its increase. More work is clearly required in order to fully understand this complex process.

In conclusion circulating levels of miR-122 and miR-200a were clearly greater at baseline in the HIV-1-infected individuals, who suffered from some degree of liver disease whilst on continuous virally suppressive ART. Additionally, these miRNAs correlated with the traditional markers of liver disease, AST and ALT and with levels of IL-6, a current marker of systemic inflammation and adverse events in ART-treated individuals^16^. MiR-122 and miR-200a represent a potential improvement on the current liver biomarkers AST and ALT. Firstly, these miRNAs may be more specific to liver disease than AST and ALT. MiR-122 is almost exclusively expressed in the liver, whereas AST and ALT can originate from a number of cellular sources, including skeletal muscle^47^. Mir-200a may not be as specific to the liver, but it’s parallel expression with miR-122 indicate that it is also originating from the liver. Secondly, the release of miR-122 and miR-200a into the circulation may be indicative of a specific cellular pathway, i.e. apoptosis, which is disrupted during liver disease, and further analysis of these miRNA may provide new avenues for therapeutics targeting this disrupted pathway. However, further work is clearly required on the exact purpose and mechanism of the release of miR-122 and miR-200a into the circulation. The data presented here, coupled with both miRNAs previous associations with liver disease in the literature, suggests that circulating miR-122 and miR-200a are promising predictive biomarkers for liver disease in the ART-treated HIV-1-infected populations. Overall, this study is hypothesis generating and these miRNAs would need to be analysed and quantified in a much larger group of HIV-1-infected before their effectiveness as liver disease biomarkers in the ART-treated population could be fully assessed. However, the data generated in this study suggests that this is an area that warrants further investigation.

## Methods

All methods in this study were performed in accordance with the relevant guidelines and regulations of the INSIGHT collaboration.

### Ethics

Samples analysed in this study were derived from participants in two international clinical trials, SMART (NCT00027352)^14^ and ESPRIT (NCT00004978)^15^, run by the INSIGHT collaboration in over 450 investigational centres between 1999 and 2009. All samples were derived from participants who provided written informed consent to use of both their data and of their stored samples for future laboratory research. All informed consents were reviewed and approved by participant site ethics review committees. The ethics for SMART, ESPRIT and this study were reviewed and approved by the UNSW Human Research Ethics Committee.

### Clinical outcomes

Causes of death among cases was reviewed by an Endpoint Review Committee and categorised using the Coding of Death in HIV (CoDE system)^48^.

### Measurement of miRNAs in serum

Detailed methodologies for the analyses of the 21 serum miRNAs, in all the cases and controls, can be found in our previous study^13^. Briefly, RNA was extracted using Trizol LS, (Life Technologies) before RNA was Reverse transcribed and pre-amplified using custom primers (Life technologies). MiRNAs were then measured in duplicate, using Taqman® primers and probes (Life Technologies), and normalised to the expression of miR-16.

### PEG-precipitation of serum particles

A new vial of frozen serum was thawed for the PEG precipitation of particles from serum (PPPs). First, 800 µl of serum was centrifuged for 10 minutes at 300xg at room temperature to remove cellular debris. This clarified serum was then filtered through a 0.22 µm filter (Merck Millipore), to remove larger biological particles, such as apoptotic bodies (approximately 1–5 µm). Following this, 450 µl of the filtered serum was added to a clean 2 mL LoBind tube (Eppendorf) and 120ul of ExoQuick reagent (Systems Biosciences, Mountain View, CA, USA) was mixed in, before being allowed to incubate for one hour at 4 °C. The mixture was then centrifuged for 30 minutes at 1500xg at room temperature. The supernatant was completely removed, and the pellet was resuspended in 100 µl of room temperature Phosphate Buffer Solution (PBS) that had been filtered using a 0.22 µM filter. A 10 µl aliquot of the resuspended particles was taken for Nanoparticle Tracking Analysis. 1 mL of Trizol LS™ (Life Technologies) was then added to the remaining purified PPP suspension for RNA extraction. All PPPs in Trizol were stored at −80 °C.

While the ExoQuick reagent is able to extract circulating vesicles efficiently, it relies on polyethylene glycol (PEG) precipitation, and does not distinguish between extra-cellular vesicles (EVs) of different sources, such as exosomes, microvesicles and apoptotic bodies. Filtering the serum with a 0.22 µm filter retains all of the exosomes (approximately 30–100 nm in size), removes the larger apoptotic bodies (approximately 1–5 µm) but does not remove all of the microvesicles (100 nm to 1 µm). Therefore, the population of EVs analysed consists of both exosomes and microvesicles, and will be referred to as PEG-purified particles (PPPs) to distinguish them from EVs as a whole.

### Nanoparticle Tracking Analysis

The 10 µl aliquot of purified PPPs was diluted 1:1 with 1% PFA in PBS and stored at 4 °C for a minimum of 1 hour in order to inactivate HIV-1^49^. This fixed solution was then diluted 1 in 10,000, to a final volume of 1 mL, in filtered PBS. This diluted sample was then run on the NanoSight NS300 machine (Malvern, Salisbury, United Kingdom), with a 405 nm laser.

The NanoSight was run using the Automatic Syringe Pump (Malvern). Flow rates for the Automatic Syringe Pump are measured in arbitrary units of speed ranging from 1–2000. Firstly, 1 mL (the maximum amount the syringe pump can process at one time) of filtered PBS was flushed through the machine at maximum speed (2000). A second aliquot of 800ul to 1 mL of filtered PBS was then flushed through the machine at maximum speed. Once the syringe had pumped all the PBS the remaining liquid in the machine was sucked back up at maximum speed. This step was performed before any new samples were run to ensure that there were no contaminating nanoparticles from previous samples. After the NanoSight had been cleaned 1 mL of the diluted sample was pushed through the machine using the syringe pump, initially using maximum speed but the rate was decreased once the particles become visible. When there was only 300 µl left in the syringe the pump was stopped in order to focus the microscope. The microscope was focused at a level that provided a clean black background (microscope level 10 or 11) and with the particles showing up clear with a slight halo around them. Once the microscope was focused, the syringe pump was restarted at a lower speed, 100–500, until 250ul was left in the syringe. The pump speed was then slowed to 50 and the standard NTA measurement of five 60 second recordings was taken. The measurements were analysed using a detection threshold of 4. Detection threshold is another arbitrary unit of measurement. A lower detection threshold will tell the software to count smaller and less bright signals as real whereas a higher detection threshold would censor these signals. NTA version 3.1 Build 3.1.45 was used for all analyses.

### RNA extraction from PPPs

RNA was extracted using TRIZOL LS (Life Technologies) according to the manufacturer’s instructions with some slight changes. Upon thawing of the PPPs in TRIZOL LS (Life Technologies) 3 µl of the cel-miR-39 spike in control (Qiagen) was added. The stock cel-miR-39 solution was diluted with dH2O (Life Technologies), to form a working solution of 1.6 × 10^8^ copies/ μl, according to manufacturer’s instructions. All other RNA extraction steps were performed according to manufacturer’s instructions.

### Reverse Transcription and Pre-Amplification of PPP RNA

#### Reverse Transcription

RNA was reverse transcribed using the Taqman MicroRNA Reverse Transcription Kit (Life Technologies). One reaction of the reverse transcription mastermix consisted of 6 µl of a custom RT primer pool (consisting of primers for miRs-122, miR-200a let-7e and CEL-miR-39, made up according to manufacturer’s instructions), 0.3 µl of dNTPs, 2ul of reverse transcriptase, 1.5 µl of 10xRT buffer, 0.19 RNase inhibitor and 2.01 µl of nuclease free water. In 96 well PCR plates 12 µl of the RT mastermix was combined with 3 µl of sample RNA. Samples were run on T100 Thermal-cycler (Biorad) for 30 minutes at 16 °C followed by 30 minutes at 42 °C and 5 minutes at 85 °C. The resulting cDNA was stored at −80 °C.

#### Pre-amplification

A single reaction of the pre-amplification mastermix used for both Taqman array cards and 384 well PCRs consisted of 12.5 µl of 2x Pre-amplification mastermix (Life Technologies) 3.75 µl of custom pre-amplification primers and 6.25ul nuclease free water. In 96 well plates 22.5 µl of the pre-amplification master-mix was combined with 2.5 µl of cDNA from the RT reaction according to the manufacturer’s instructions. Samples were run on a Veriti Thermocycler (Life Technologies) for 95 °C for 10 minutes, 2 minutes at 55 °C, 2 minutes at 72 °C followed by 12 cycles of (15 seconds at 95 °C and 4 minutes at 60 °C) and finally 99.9 °C for 10 minutes. The resulting pre-amplification product was diluted 1:8 with 0.1x TE buffer and stored at −80 °C.

### RTqPCR

RTqPCR was performed for miRs-122, miR-200a, let-7e and cel-miR-39 in 96 well plates on the Life Technologies Quantstudio7 machine according to manufacturer’s instructions. Briefly; one PCR reaction contained 0.5 µl of Taqman® primer/probe mix (Life Technologies), 10 µl of 2x Gene Expression Master-mix (Life Technologies), 6.5 µl of DNase/RNase free H2O and 3ul of diluted pre-amplification product.

For miR-200a and let-7e if cel-miR-39 and miR-122 was detected < 30 cycles in a sample but miR-200a or let-7e was not detected the sample was designated as having a Ct of 35 (the lower limit of detection) to signify extremely low expression or no expression of that particular miRNA. If cel-miR-39 and/or miR-122 was detected over 30 cycles and either miR-200a or let-7e wasn’t detected the RNA or extraction was considered unreliable and this sample was not used in further analyses.

### Statistics

Relative expression of miRs-122, -200a and let-7e from the serum was calculated using the Equation 1 (listed below):

1 $${2}^{-({\rm{Ct}}({\rm{miRNA}}{\rm{of}}{\rm{interest}})-{\rm{Ct}}({\rm{miR}} \mbox{-} 16))}$$

Relative expression of miRs-122, -200a and let-7e from the purified PPPs was calculated using Equation 2 (listed below):

2 $${2}^{-({\rm{Ct}}({\rm{miRNA}}{\rm{of}}{\rm{interest}})-{\rm{Ct}}({\rm{CEL}}-{\rm{miR}} \mbox{-} 39))}/{\rm{PPP}}\,{\rm{concentration}}$$

Statistical analyses were performed using Prism® (Graphpad Software Inc, La Jolla, USA). Comparisons between the HCV/HIV co-infected individuals and HIV-monoinfected were performed using a multiple t test with a Sidak-Bonferroni corrected p value, with a result deemed significant with p < 0026. All other comparisons between cases and controls were are uncorrected for multiple testing and were performed using a Mann-Whitney U test with a result deemed significant with p < 0.05. Correlations between the miRNAs and PPP count, AST and ALT were assessed using Spearman’s non-parametric correlation coefficient with a relationship deemed significant with p < 0.05. Levels of miRNAs, PPP count, AST and ALT were then log_10_ normalised and plotted on xy scatter plots using Prism®.

## Electronic supplementary material

Supplemental Information
