# Supplementary material for: Circulating miR-122 and miR-200a as biomarkers for fatal liver disease in ART-treated, HIV-1-infected individuals
Source: Sci Rep. 2017 Sep 7;7:10934. doi: 10.1038/s41598-017-11405-8 (PMC5589757; doi:10.1038/s41598-017-11405-8)
Supplement: Supplementary file 1 — Supplemental Information [file 41598_2017_11405_MOESM1_ESM.doc]

# Supplemental Information

**Circulating miR-122 and miR-200a as biomarkers for fatal liver disease in ART-treated, HIV-1 infected individuals**

Daniel D Murray*^1^, Kazuo Suzuki^1^, Matthew Law^1^, Jonel Trebicka^2^, Jacquie Neuhaus Nordwall^3^, Margaret Johnson^4^, Michael J. Vjecha^5^, Anthony D Kelleher^1^, Sean Emery^1,6^ and the INSIGHT ESPRIT and SMART Study Groups

^1^ The Kirby Institute for Infection and Immunity in Society, University of New South Wales, Sydney, Australia

^2^ Department of Internal Medicine, University of Bonn, Bonn, Germany

^3^ University of Minnesota, Minneapolis, Minnesota, United States of America

^4^ Ian Charleson Day Centre, Royal Free Hampstead NHS Trust, London, United Kingdom.

^5^ Institute for Clinical Research, Veterans Affairs Medical Center, Washington D.C., United States of America.

^6^ Faculty of Medicine, University of Queensland, Brisbane, Australia

* Corresponding author

E-mail: [dmurray@kirby.unsw.edu.au](mailto:dmurray@kirby.unsw.edu.au)

Supplemental Table 1.

| **Baseline Characteristics** | **Case (n=126)** | **Control (n=247)** |
| --- | --- | --- |
| **Age ± SD (years)** | 48.29 ± 10.60 | 47.29 ± 9.62 |
| **Mean baseline CD4+ T cell count ± SD** | 533.865 ± 224.37 | 584.94 ± 253.11 |
| **Mean nadir CD4+ T cell count± SD** | 207.04 ± 159.18 | 232.79 ± 172.90 |
| **Mean BMI ± SD** | 24.78 ± 5.36 | 24.84 ± 4.13 |
| **Mean hs-CRP ± SD** | 5.62 ± 8.53 | 3.97 ± 8.56 |
| **Mean D-dimer ± SD** | 0.50 ± 0.54 | 0.41 ± 0.68 |
| **Mean IL-6 ± SD** | 5.03 ± 10.11 | 2.67 ± 2.36 |
| **%male** | 82.54 | 82.52 |
| **Race (%white)** | 73.81 | 69.51 |
| **Race (%black)** | 19.84 | 19.92 |
| **Race (%other)** | 6.35 | 10.57 |
| **Off ART (%)** | 10.32 | 7.72 |
| **On ART with HIV RNA ≤500 (%)** | 61.90 | 74.39 |
| **On ART with HIV RNA >500 (%)** | 27.78 | 17.89 |
| **HBV (% surface antigen positive)** | 3.97 | 2.44 |
| **HCV (% antibody positive)** | 30.95 | 17.48 |
| **Prior AIDS (% baseline positive)** | 29.37 | 26.02 |
| **Diabetes (% baseline positive)** | 8.73 | 4.47 |
| **Prior CVD (% baseline positive)** | 8.73 | 1.63 |
| **Lipid Lowering Drug (% baseline positive)** | 18.25 | 17.48 |
| **Blood Pressure Lowering Drug (% baseline positive)** | 23.02 | 12.6 |
| **Smoking (% smokers)** | 59.32^1^ | 36.75^1^ |
| **Mean total cholesterol ± SD** | 195.2 ± 53.21^1^ | 198.8 ± 44.57^1^ |
|  |  |  |

^1^ Details were available for the SMART study only; control n=117, case n=59

## Supplemental Figure Legends


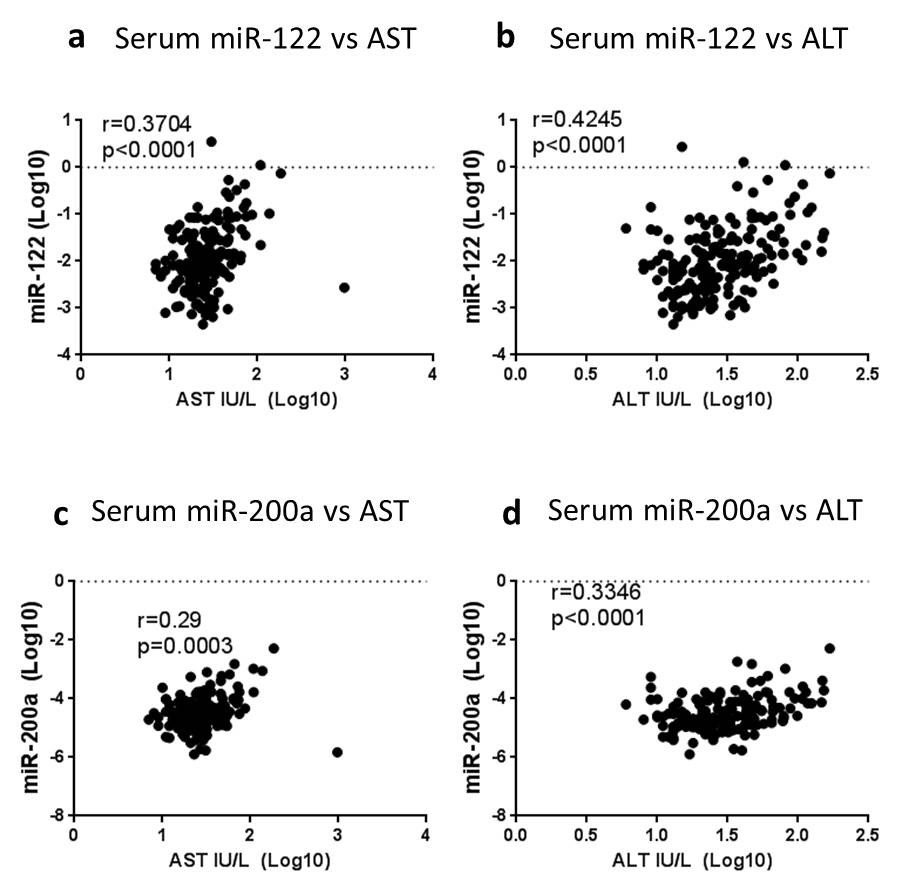


Supplemental Figure 1. Serum miR-122 and miR-200a correlations with AST and ALT.

Relative expression (normalised to miR-16) of miR-122, miR-200a and let-7a, measured in the serum of the all 373 cases and controls (from our previous study ^1^), and AST/ALT were log normalised and plotted. Correlations were analysed using Spearman’s non-parametric correlation coefficient with a relationship deemed significant with p<0.05. Matched XY pairs were available for 178 individuals in the miR-122 vs AST analysis, 154 in the miR-200a vs AST, 180 in the miR-122 vs ALT and 160 for miR-200a vs ALT.


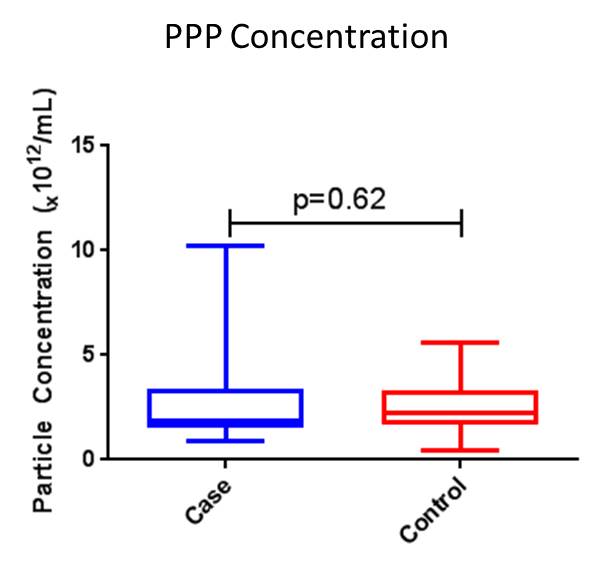


Supplemental Figure 2. Concentration of PPPs in the liver cases and controls.

Particle concentrations were measured using NanoSight tracking analysis software on the Malvern NanoSight 300. There was no difference in total PPP concentration between liver cases and controls. Differences were measured using a Mann-Whitney T test with differences deemed significant with p<0.05.


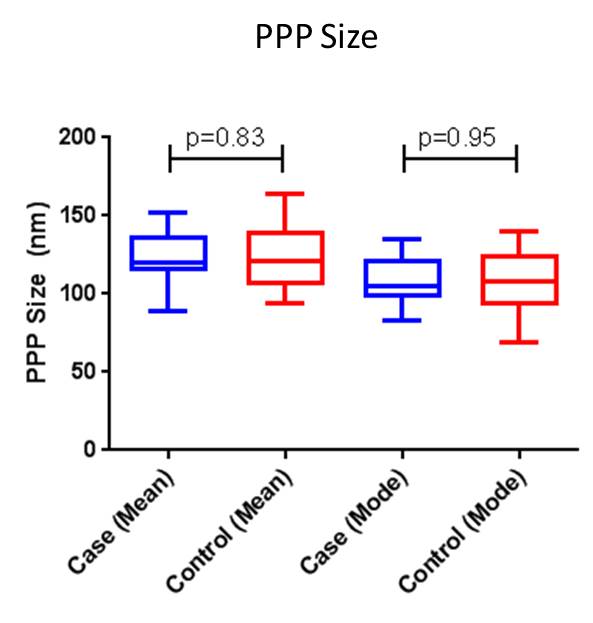


Supplemental Figure 3. Size of the PPPs in the liver cases and controls. Nanoparticle

Tracking Analysis also measures the size (mean and mode) of particles it detects. However, there was no difference between liver cases and controls in both mean and mode of PPP size. Differences were measured using a Mann-Whitney T test with differences deemed significant with p<0.05.


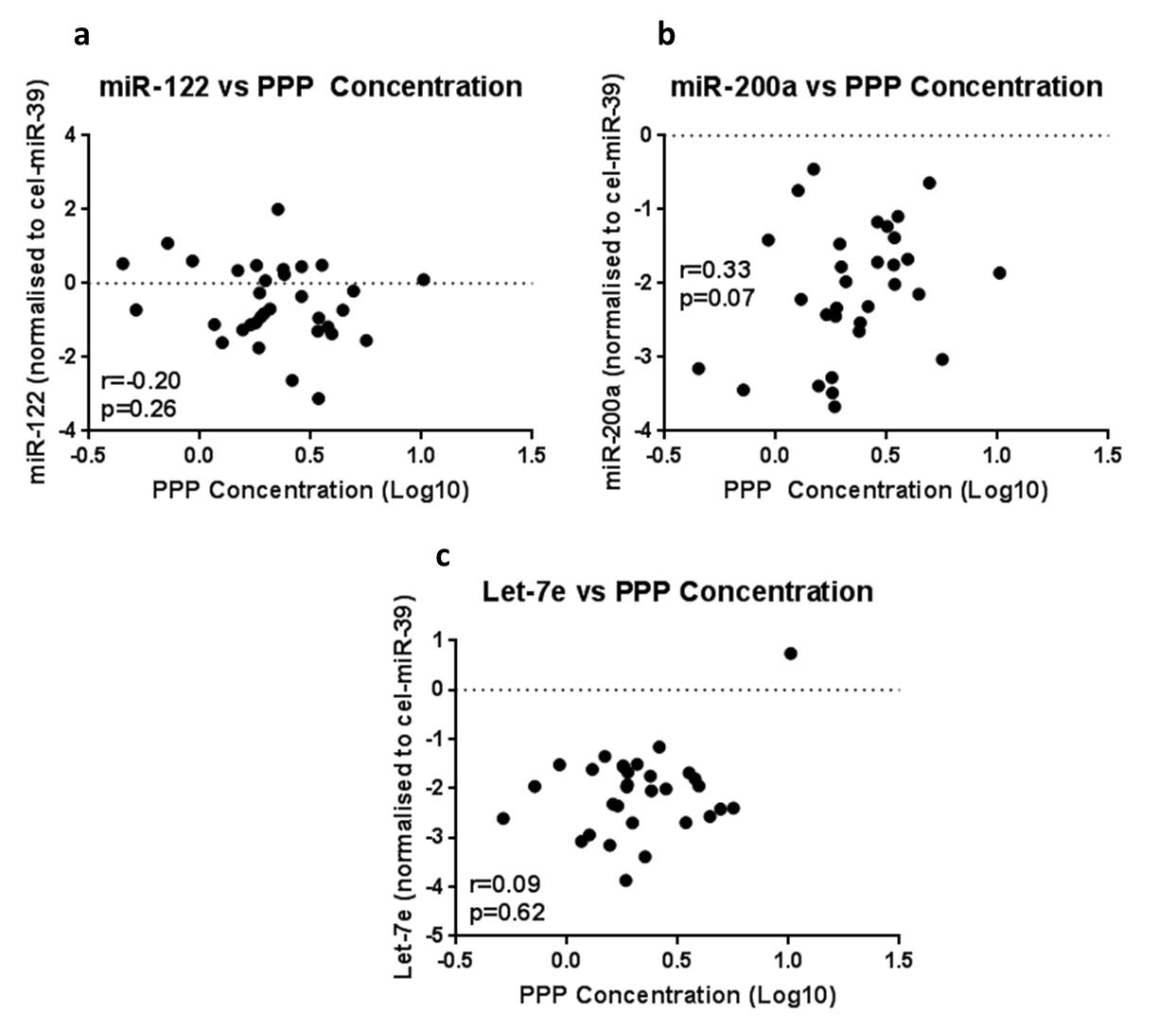


Supplemental Figure 4. Correlations between the PEG-purified particle (PPP) associated miRNAs and the PPP concentrations. No miRNAs measured in the PPPs were significantly correlated with PPP concentration. miRNA values are represented as Log10 transformed values, normalised to cel-miR-39. Correlations were analysed using Spearman’s non-parametric correlation coefficient with a relationship deemed significant with p<0.05.

# Supplemental References

1 Murray, D. *D. et a*l. Circulating microRNAs in Sera Correlate with Soluble Biomarkers of Immune Activation but Do Not Predict Mortality in ART Treated Individuals with HIV-1 Infection: A Case Control Study*. PLoS O*n**e** 10, e0139981, doi:10.1371/journal.pone.0139981 (2015).
